# Autophagy as a potential link between progesterone responsiveness and endometrial immune function in recurrent implantation failure

**DOI:** 10.3389/fimmu.2026.1990439

**Published:** 2026-10-12

**Authors:** Li Guo, Jinghong Xie, Mengyu Wang, Shuang Ma, Xinyan Zhang, Yu Guan

**Affiliations:** Xiyuan Hospital, China Academy of Chinese Medical Sciences, Beijing, China

**Keywords:** autophagy, decidualization, endometrial immune microenvironment, progesterone resistance, recurrent implantation failure, uterine natural killer cells

## Abstract

Recurrent implantation failure (RIF) describes a clinical outcome rather than a single diagnosis or immune disorder. During progesterone-driven decidualization, changes in stromal metabolism and epithelial secretion accompany the production of signals that support uterine natural killer (uNK) cell residency. Autophagy contributes to these changes, although its role in RIF is unresolved. Genetic studies in mice and human stromal cells implicate autophagy-related proteins in progesterone responses and decidual differentiation. Some RIF tissue studies, however, report increased autophagy-associated signals. Increased flux accompanies impaired progesterone responses in some stromal models and improved decidualization in others. These contrasting findings may reflect the need to maintain autophagic activity within a suitable range for each cell compartment and cycle phase, rather than a uniform deficit. Progesterone-responsive stromal cells release interleukin 15 (IL-15) and related signals that recruit and program uNK cells. Their residency may also depend on epithelial secretion, adhesion, and matrix remodeling. In cycling human endometrium, activated uNK cells contribute to tissue quality control by clearing senescent maternal decidual cells; the embryo is not the target of this process. Single-cell and spatial studies of RIF endometrium have identified altered epithelial, stromal, and NK states. Whether progesterone resistance causes these cellular changes and, in turn, implantation failure remains unknown. This review examines the evidence for these relationships and the experiments needed to assess autophagic flux beyond static markers and uNK function beyond cell abundance. Current evidence does not support routine NK testing or autophagy-directed treatment in RIF.

## 1 Introduction

The number of unsuccessful embryo transfers is often used to describe recurrent implantation failure (RIF) in clinical practice. The European Society of Human Reproduction and Embryology (ESHRE) defines it within assisted reproduction as failure to achieve a positive pregnancy test after the transfer of embryos judged to be viable, when the cumulative likelihood of implantation warrants further investigation. A cumulative predicted implantation probability above 60% has been proposed as the threshold for further work-up (1). Under this definition, RIF occurs before a biochemical pregnancy is achieved. Recurrent pregnancy loss (RPL) follows implantation and subsequent pregnancy failure (1). Studies of RPL, first-trimester decidua, and abortion-prone mice may suggest mechanisms relevant to RIF. Because they examine events after implantation, however, their findings require separate interpretation from those in implantation failure. Endometrial dysfunction is a possible contributor to unexplained RIF after impaired embryo competence, transfer-related factors, and recognizable uterine or systemic causes have been excluded. The underlying endometrial changes are heterogeneous (1). This Mini Review considers autophagy as a potential link between progesterone responsiveness, stromal decidualization, epithelial receptivity, and local uterine natural killer (uNK) cell support (**Figure 1**). The proposed connections have not been demonstrated as a causal pathway in patients. Protein or transcript abundance alone does not measure autophagic flux (2, 3), and cell counts do not measure cell function (4). The natural killer (NK) populations also differ. Studies of uNK cells in cycling endometrium, decidual NK (dNK) cells after implantation, and peripheral-blood NK (pNK) cells examine distinct populations (4), which may partly explain conflicting results.

**Figure 1:**
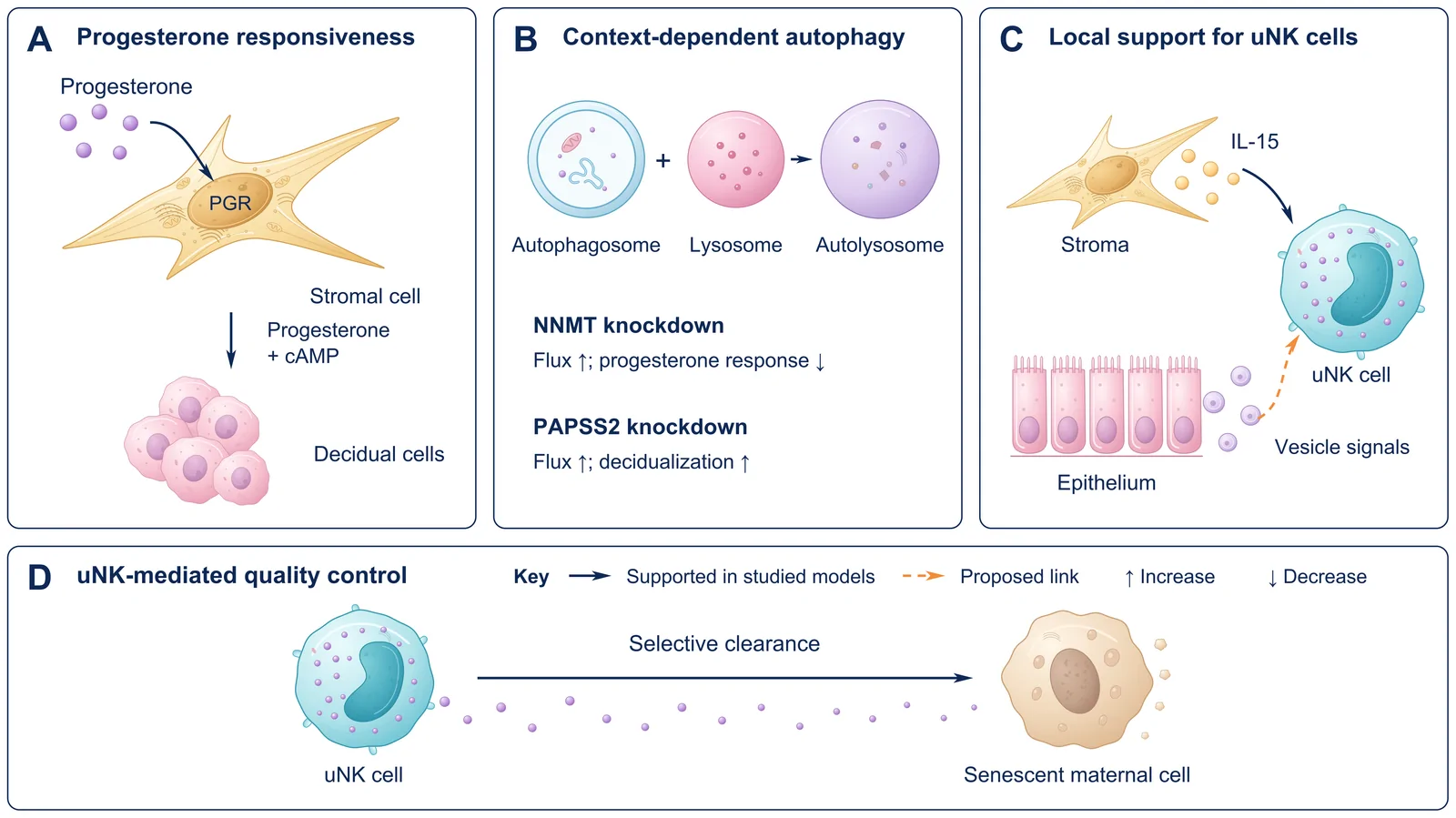
Proposed links among progesterone responsiveness, autophagy, and uNK biology in RIF. **(A)** Progesterone- and cAMP-supported stromal decidualization. **(B)** Different outcomes associated with increased flux after NNMT or PAPSS2 knockdown. **(C)** Stromal IL-15 signaling and a proposed epithelial vesicle link to uNK cells. **(D)** Selective clearance of senescent maternal decidual cells by uNK cells. Solid arrows indicate processes supported in the stated models; the dashed arrow indicates a proposed link. The panels do not represent an established causal sequence in RIF. Evidence for autophagy-dependent dNK retention comes from studies after implantation.

## 2 Progesterone resistance and defective decidualization

Progesterone resistance refers to an attenuated, mistimed, or qualitatively abnormal response to an adequate progesterone signal. Progesterone resistance is distinct from low circulating progesterone. An isolated reduction in progesterone receptor (PGR), prolactin (PRL), or insulin-like growth factor-binding protein 1 (IGFBP1) is also insufficient to identify progesterone resistance. The concept derives largely from endometriosis, in which impaired responsiveness can occur despite apparently normal hormone levels (5). In response to progesterone and rising cyclic adenosine monophosphate (cAMP), stromal fibroblasts exit the cell cycle and undergo transcriptional, metabolic, secretory, and matrix-remodeling changes. PRL and IGFBP1 reflect parts of this response but do not directly measure decidual competence (6, 7). In a study of nicotinamide N-methyltransferase (NNMT), knockdown reduced PGR, homeobox A10 (HOXA10), and heart and neural crest derivatives expressed 2 (HAND2). PRL and IGFBP1 remained unchanged (8). The change in progesterone responsiveness would have been missed if only PRL and IGFBP1 had been measured. Decidualization also produces mature cells alongside an acutely senescent subset, and bulk measurements do not capture the balance between these populations that underlies tissue quality (9).

Forkhead box O1 (FOXO1), HAND2, and their associated PGR networks coordinate this transition (6, 10). In human stromal cells, HAND2 binds a CCTCTGG motif at -1628/-1622 upstream of the interleukin 15 gene (IL15) and drives its transcription (10). This provides a possible route from hormonal differentiation to local uNK support, but the effect of HAND2 on IL15 transcription was modest compared with estradiol plus progestin (10). Other regulators probably contribute to IL-15 output. The direction of IL15 regulation also depends on stromal cell state (11). It remains unclear whether disruption of the HAND2-IL-15 pathway causes RIF.

Progesterone-response defects have been reported in RIF, but the findings are inconsistent. Endometrial gland organoids derived from RIF patients showed diminished responses to sex steroids (12). Single-cell and spatial studies also identify altered cell states that vary by cell population and tissue region (13). This variation makes progesterone resistance difficult to characterize with a single bulk marker across patients.

Autophagy and progesterone responsiveness appear to influence one another during decidualization. In mice, Pgr-Cre-mediated loss of focal adhesion kinase family-interacting protein of 200 kDa (FIP200) caused sustained uterine epithelial proliferation despite adequate progesterone. Implantation sites were absent by 5 days post coitum (dpc), and decidualization failed. Knockdown of RB1CC1, which encodes FIP200, reduced PRL and IGFBP1 induction in human cells (14), as did depletion of autophagy-related proteins 5 or 7 (ATG5 or ATG7) (15). These perturbation experiments leave open whether deficient flux is the main defect in human RIF. ATG proteins have non-autophagic functions (2). Several RIF tissue studies report increased autophagy-associated signals (8, 16). Cell type, timing, and stress may help account for these differences.

## 3 Context-dependent autophagy in endometrial receptivity

The contrasting stromal findings suggest that more autophagy does not necessarily improve decidualization (8, 17). A single measurement of autophagosome number, microtubule-associated protein 1 light chain 3 (LC3) lipidation, or sequestosome 1 (SQSTM1/p62) cannot distinguish increased cargo delivery from impaired clearance. Autophagosomes can accumulate because formation has accelerated, degradation has slowed, or both. Distinguishing these possibilities requires time-resolved measurements, lysosomal blockade, or tandem fluorescent reporters, ideally combined with genetic perturbation (2, 3). Without a measurement of degradative throughput, fixed-biopsy findings are best described as autophagy-associated markers. Autophagy-associated signals also vary with hormonal state, cell lineage, and sampling time (18). Molecular autophagy should also be distinguished from the broader group of autophagy-related processes (19).

Experimental studies implicate autophagy machinery in stromal differentiation. Autophagic activity increases as human stromal cells decidualize, and ATG5 or ATG7 depletion reduces PRL and IGFBP1 induction (15). FIP200/RB1CC1 deficiency produced similar defects in mouse uterine tissue and human cells (14). Conditional loss of Atg16L1 and depletion of ATG9A, the latter accompanied by impaired mitophagy, also caused decidual defects (20, 21). The targeted proteins are required in these models, but the contribution of their autophagic functions is less clear. FIP200, ATG5, ATG7, and ATG16L1 also have non-autophagic roles documented in other experimental systems (2, 22). Targeted rescue or separation-of-function experiments could resolve which effects depend on autophagy.

Several clinical RIF studies report increased autophagy-associated signals. Endometrium from patients with unexplained RIF showed higher LC3-II, lysosome-associated membrane protein 2 (LAMP2), and hypoxia-inducible factor 1-alpha (HIF-1alpha), together with lower p62 (16). RIF with chronic endometritis has also been associated with changes in inflammatory mediators and autophagy-related markers (23). Both studies were cross-sectional and relied largely on static measurements, in which even the combination of high LC3-II and low p62 cannot establish the direction of flux (2, 3). A separate study of mid-secretory RIF endometrium found reduced Notch receptor 1 (NOTCH1) signaling, increased autophagosome abundance by electron microscopy, and lower receptivity markers. NOTCH1 knockdown in Ishikawa cells reproduced this phenotype through autophagy linked to protein kinase B (AKT) and mechanistic target of rapamycin (mTOR) signaling (24). Flux was not measured in the patient tissue. The use of a carcinoma line, Ishikawa cells, also limits extrapolation to receptive epithelium.

The NNMT study combined complementary flux assays with analysis of mid-luteal RIF endometrium, where NNMT expression was reduced. Its knockdown in decidualizing stromal cells increased flux and reduced PGR, HOXA10, and HAND2 (8). Bafilomycin A1 treatment and a tandem LC3 reporter combining mCherry with enhanced green fluorescent protein (eGFP) supported the authors’ interpretation of increased flux. Recombinant aldehyde dehydrogenase 1 family member A3 (ALDH1A3) partially rescued the autophagy and progesterone phenotypes, and uterine NNMT inhibition reduced implantation sites in mice (8). Without autophagy-specific epistasis experiments, it is unclear whether increased flux causes the progesterone-response defect or accompanies it.

Zi et al. compared 8 RIF and 10 control endometrial samples collected 5–7 days after ovulation from women aged 20–35 years. RIF required failure to conceive after at least three transfers involving four or more good-quality embryos; controls conceived after their first transfer for male-factor infertility. Stromal sulfotyrosine staining was higher in RIF (17). The authors studied 3’-phosphoadenosine 5’-phosphosulfate synthase 2 (PAPSS2) in telomerase-immortalized human endometrial stromal cells (T-HESCs) and primary stromal cells from control donors. Decidualization was induced with progesterone and dibutyryl-cAMP (17).

PAPSS2 knockdown reduced sulfation of bone morphogenetic protein receptor type 1A (BMPR1A), increased its binding to bone morphogenetic protein 2 (BMP2), and enhanced SMAD family member 1/5/9 (SMAD1/5/9) phosphorylation. BMPR1A re-expression experiments with the Y457F mutant supported a role for receptor sulfation (17). After PAPSS2 knockdown, a tandem green/red fluorescent LC3 reporter showed more red-only puncta, accompanied by reduced p62. These findings supported increased flux, whereas PAPSS2 overexpression produced the opposite pattern. LDN193189, a BMP receptor inhibitor, and 3-methyladenine attenuated the gains in decidual markers and BeWo spheroid spreading. In ovariectomized mice, the BMP signaling agonist BMPSB4 enhanced oil-induced decidualization, and 3-methyladenine partly reversed this effect (17).

Increased flux appeared beneficial under these experimental conditions. The tissue comparison was small and observational, however, and neither BMPR1A sulfation nor autophagy markers were measured directly in RIF biopsies. Autophagy dependence was assessed by pharmacological inhibition, without genetic autophagy rescue, while the mouse interventions targeted BMP signaling rather than PAPSS2 itself. BeWo spreading and artificial decidualization are surrogate endpoints, and uNK function was not tested. The study therefore does not establish improved implantation or live birth in RIF, or a general benefit from activating autophagy (17).

Endometrial function may depend on maintaining autophagic activity within an appropriate range rather than maximizing or suppressing it. Impaired mitophagy may restrict mitochondrial remodeling during stromal differentiation (21). Increased flux can also accompany reduced progesterone responsiveness, as observed after NNMT knockdown (8). Single-cell RIF data identified lower proportions of a PGR-high epithelial population enriched for autophagy and exosome programs and a resident NK subset. Autophagy was not perturbed in that study (25). Phase-matched stromal and epithelial systems derived from patients, including hormone-responsive endometrial organoids, could help test these relationships (26). Flux assays combined with inhibitors, graded perturbations, and rescue at multiple steps could be used before and during decidualization. For therapeutic interpretation, measurements of overall autophagy would need to be placed in the context of cell compartment, cycle timing, and subtype.

## 4 Potential roles of stromal and epithelial autophagy in uNK regulation

Because epithelial, stromal, and immune cells change differently over the menstrual cycle, the interpretation of autophagy-associated signals depends on biopsy timing and cellular origin (27, 28). Reference maps and continuous timing estimates help distinguish differences in tissue composition from variation in biopsy timing (29, 30). Progesterone- and cAMP-driven decidualization alters the signals that stromal cells provide to local immune cells (9, 10). The HAND2-IL15 relationship depends on cell state: depleting HAND2 in proliferative fibroblasts increased IL15-dependent NK migration (11). The direction of the HAND2 effect on IL15 may therefore differ between stromal states.

Local signals influence NK-cell recruitment, retention, survival, and adaptation to the tissue. During both endometrial regeneration and pregnancy, human uterine NK cells differentiate along a continuum driven by progesterone-regulated stromal IL-15 (31). In early pregnancy, trophoblast-derived C-X-C motif chemokine ligand 12 (CXCL12) recruited C-X-C motif chemokine receptor 4 (CXCR4)-bearing CD16-negative NK cells, and IL-15 exposure altered part of their chemokine-receptor repertoire (32). Eomesodermin (EOMES)-positive subsets in the uterus and decidua expressed tissue-associated receptors that could be partly induced by local signals (33). Whether these early-pregnancy findings apply before implantation is unknown, and CD56 counts alone cannot distinguish changes in recruitment, retention, survival, or reprogramming.

Experiments in early-pregnancy decidua link stromal autophagy to NK localization, but this relationship has not been established in preimplantation RIF. Decidual stromal-cell autophagy increased adhesion molecules and promoted dNK adhesion and retention through melanocyte inducing transcription factor (MITF), tumor necrosis factor receptor superfamily member 14 (TNFRSF14; also called herpesvirus entry mediator, HVEM), and matrix metalloproteinase 9 (MMP9). Perturbing stromal ATG5 or TNFRSF14 altered this interaction (34). By contrast, treating dNK cells with 3-methyladenine did not significantly alter their adhesion in the tested assay. Low-dose rapamycin also reduced embryo resorption in abortion-prone mice (34). Because these experiments used dNK cells and miscarriage models, their relevance to RIF needs to be tested in the appropriate cell populations before implantation.

Epithelial regulation of uNK cells is supported mainly by associations. Single-cell profiling of roughly 60,000 cells from six RIF patients and three fertile controls found lower proportions of CD63-high/PGR-high epithelial cells and CD49a-positive/CXCR4-positive uNK cells. The epithelial population expressed progesterone-response, autophagy, and exosome transcripts. Blocking exosome release with GW4869 altered the NK phenotype in co-culture (25). The nine donors formed the single-cell cohort; additional samples were used for tissue validation (25). The co-culture perturbation was not specific to autophagy, so it cannot establish the proposed pathway, which remains unproven *in vivo*. Reduced NOTCH1 signaling with increased autophagy was also associated with impaired epithelial receptivity, but this study used a carcinoma line and did not assess uNK cells (24).

The stromal findings differ according to the signaling pathway studied. NNMT deficiency increased flux while disrupting progesterone signaling, whereas reducing PAPSS2 increased BMP2-SMAD-dependent flux and improved decidualization (8, 17). Trophoblast autophagy can also modulate dNK activation after implantation, although this evidence concerns the fetal compartment (35). Testing whether autophagy coordinates local NK support would require epithelium, stroma, and uNK cells from the same patients at a matched cycle phase. Perturbing flux separately in each compartment could then be paired with measurements of IL-15, chemokines, extracellular vesicles, adhesion, residence, and uNK function. Until such interactions are tested directly, this coordinating role remains a hypothesis.

## 5 uNK dysregulation and failed decidual quality control

Uterine NK cells are tissue-adapted lymphocytes that accumulate during the secretory phase and have phenotypes distinct from those of paired pNK cells (36, 37). Across nearly two thousand biopsies timed to the luteinizing hormone (LH) surge, uNK abundance increased roughly threefold between 5 and 12 days after the surge (9). Function also varies across the cycle, with all three uNK subsets showing the greatest degranulation and cytokine production during the secretory phase (38). Pregnancy is associated with further dNK specialization (39, 40). Single-cell surveys place these cells within the decidual immune environment (41), and recent reviews describe several uNK subpopulations (42). These pregnancy-associated states need to be considered separately from those in cycling, non-pregnant endometrium.

Decidual quality control is one of the best-supported functions of uNK cells in cycling endometrium. Stromal differentiation produces mature decidual cells and an acutely senescent, pro-inflammatory subpopulation (9). IL-15-activated uNK cells remove the senescent cells through granule exocytosis, allowing stromal differentiation to continue. Granzyme B inhibition blocks this clearance (9). The targets in this process are senescent maternal cells. The effect of senescence also depends on its duration. In endometrial assembloids that contained no uNK cells, transient senescent states supported implantation-associated signaling, whereas persistent senescence destabilized the tissue (43). Separate stromal studies linked persistent senescence to impaired decidualization and defective trophoblast interaction, but did not directly test uNK clearance (44). These models suggest that both the timing and persistence of senescence influence decidual function.

Signals from the embryo may also regulate the clearance of senescent decidual cells. High-molecular-weight hyaluronan inhibited CD44-dependent uNK clearance of senescent decidual cells. Medium conditioned by embryos that failed to implant also inhibited clearance, and recombinant hyaluronidase 2 (HYAL2) reversed that inhibition. Blastocyst-secreted HYAL2 levels correlated with morphological score and implantation potential (45). These findings are consistent with endometrial sensing of embryo quality (46), with maternal decidual cells as the targets of clearance rather than the embryo. Further evidence linking stromal senescence to reproductive failure comes from RPL studies. Peri-implantation endometrium showed a pro-senescent decidual response (47), and dNK-derived tumor necrosis factor superfamily member 14 (TNFSF14) restrained decidual senescence after implantation (48). The same mechanisms have not been established in RIF.

Studies of RIF report different stromal and immune abnormalities. A study of 37 RIF patients and 71 fertile controls found lower stromal cell division cycle 42 (CDC42) expression in RIF. Experimental CDC42 knockdown induced stromal senescence and decidualization defects through AKT, glycogen synthase kinase 3-beta (GSK3beta), and beta-catenin signaling. The study did not test NK involvement (49). A separate single-cell study of six RIF patients and three controls showed a lower proportion of CD49a-positive/CXCR4-positive uNK cells (25). Time-series profiling combined 18 fertile-control biopsies across LH + 3 to LH + 11 with 10 RIF biopsies at LH + 7. It identified a timing-displaced RIF class and a hyperinflammatory class with elevated interferon gamma (IFNG) expression in NK and T cells (50). Spatial analysis found region-specific transcriptional changes within CD56-positive areas but did not directly test uNK function (13). RIF may therefore encompass several endometrial abnormalities rather than one consistent immune defect.

The clinical value of uNK counts is also uncertain because abundance does not describe function. Endometrial uNK counts did not predict implantation in a timed *in vitro* fertilization (IVF) cohort (51). A meta-analysis found modestly higher endometrial CD56-positive cell levels in RIF, with a standardized mean difference of 0.49 and I-squared of 84%. Pooled analyses of the included recurrent miscarriage and RIF cohorts found no significant difference in pregnancy outcomes by uNK level. Endometrial and pNK counts were also not significantly correlated (4). A group-level difference in uNK counts is therefore insufficient to establish their predictive value in individual patients. Exploratory analyses of a small cohort with mixed reproductive failure suggested lower uNK receptor expression, unstimulated degranulation, and stimulated tumor necrosis factor (TNF) production. These uNK differences were not significant after Holm-Bonferroni correction for multiple comparisons (52). Cycle phase and stimulation conditions need to be standardized in functional assays: uNK responses vary across the cycle and between unstimulated and stimulated measurements (38, 52). First-trimester dNK cells regulate trophoblast invasion and produce angiogenic factors (53, 54). Their baseline cytotoxicity is limited, and their activating synapses are immature (55). These functional observations do not support an embryo-attack model (56, 57). ESHRE does not recommend routine NK-cell testing in RIF (1).

## 6 Discussion: translational opportunities and evidence gaps

Differences in study design and biological setting limit how these findings can be interpreted clinically. RIF biopsy studies differ in cohort definition and sampling time (1), as well as the endometrial compartments examined (**Table 1**). Spatial profiling can reveal regional changes obscured by bulk analysis (13). Continuous timing estimates can address uncertainty in biopsy dates based on urinary ovulation tests (30). Single-cell sequencing can resolve cell states but does not measure autophagic flux. Static LC3/p62 measurements also cannot distinguish changes in autophagosome formation from changes in lysosomal turnover (2). Cell-line and mouse experiments can identify mechanisms within defined systems, leaving their prevalence in patients uncertain. First-trimester decidua, miscarriage cohorts, and embryo-resorption models also concern postimplantation biology; mechanisms relevant to RPL cannot be assumed to explain RIF.

**Table 1:** Evidence summary for the proposed progesterone-autophagy-uNK framework.

Model/context	Mechanism/observation	Functional readout	Key limitation	Ref.
Human endometrial stromal cells; *in-vitro* decidualization	Autophagic activity rose during decidualization; ATG5 or ATG7 depletion perturbed the pathway.	PRL and IGFBP1 induction fell after ATG depletion.	Immortalized cells; ATG proteins have non-autophagic functions; ATG5 and ATG7 effects were not identical.	(15)
Pgr-lineage Fip200-deficient mouse uterus and human stromal cells	FIP200/RB1CC1 linked the progesterone response to uterine receptivity and decidualization.	No implantation sites at 5 dpc, sustained epithelial proliferation despite progesterone, and blunted PRL/IGFBP1 after human RB1CC1 knockdown.	Broad Pgr-Cre lineage; FIP200 is not exclusively autophagic; no RIF cohort.	(14)
Mouse uterus and cultured human stromal cells	ATG16L1-dependent machinery and ATG9A-dependent mitophagy supported decidual differentiation.	Impaired decidual response or reduced differentiation markers after knockout or knockdown.	Models and targets differ; no human RIF validation; genetic effects need rescue and pathway-specific controls.	(20, 21)
RIF endometrium, decidualizing stromal cells, and a mouse model	NNMT loss was associated with increased flux as interpreted from bafilomycin A1 blockade and an mCherry-eGFP-LC3 reporter, alongside H3K9me3-mediated ALDH1A3 repression.	Lower PGR, HOXA10 and HAND2 with PRL and IGFBP1 unchanged; partial rescue by recombinant ALDH1A3; fewer implantation sites after uterine NNMT inhibition.	No autophagy-specific epistasis establishes increased flux as the necessary mediator; human data are cross-sectional.	(8)
Unexplained RIF endometrial tissue	Higher LC3-II, LAMP2 and HIF-1alpha; lower p62; increased autophagy-associated puncta.	Cross-sectional molecular and tissue-marker differences.	Static measurements do not establish flux; no mechanistic perturbation or uNK functional readout.	(16)
Mid-secretory RIF tissue and Ishikawa cells	Reduced NOTCH1 signaling and more tissue autophagosomes; NOTCH1 knockdown activated AKT-mTOR-linked autophagy in Ishikawa cells.	Reduced tissue receptivity markers and impaired cell-line receptivity after NOTCH1 knockdown.	No direct flux measurement in patient tissue; Ishikawa is a carcinoma line; uNK function was not assessed.	(24)
8 RIF/10 control mid-secretory samples; T-HESCs and control-donor primary stromal cells; BeWo spheroids; ovariectomized mice.	PAPSS2 knockdown reduced BMPR1A sulfation and enhanced BMP2-SMAD1/5/9 signaling. Tandem LC3 reporter and p62 supported increased flux.	Increased decidual markers and BeWo spreading; LDN193189 and 3-methyladenine attenuated these effects. BMPSB4 enhanced artificial decidualization (5 mice/group).	Small observational tissue comparison; no BMPR1A sulfation or autophagy profiling in RIF biopsies. Pharmacological autophagy inhibition; no autophagy gene rescue, clinical outcomes, or uNK assays.	(17)
Mid-window RIF single-cell atlas (6 RIF vs 3 controls) and epithelial-NK co-culture	Lower proportions of a CD63-high/PGR-high epithelial state carrying autophagy and exosome signatures and a CD49a+CXCR4+ NK state.	Exosome inhibition with GW4869 shifted the NK phenotype in co-culture.	Nine donors in the single-cell cohort; transcript signatures are not flux; the co-culture perturbation was not autophagy-specific.	(25)
Spontaneous-abortion decidua, stromal-dNK adhesion assays, and mice	Stromal autophagy promoted dNK adhesion and retention through a MITF-TNFRSF14/HVEM-MMP9 program.	Altered dNK residence and pregnancy outcome; low-dose rapamycin reduced embryo resorption.	Postimplantation disease; dNK rather than cycling uNK. 3-Methyladenine treatment of dNK cells did not significantly alter adhesion in the tested assay. Rapamycin is pleiotropic.	(34)
Cycling human endometrium and stromal-uNK co-culture	HAND2 bound an IL15 upstream motif at -1628/-1622 and induced transcription; IL-15-activated uNK cells recognized senescent decidual cells.	IL15 transcription; granzyme-dependent clearance of senescent maternal cells; uNK density up roughly threefold from LH + 5 to LH + 12.	Separate experiments; HAND2 induction is modest beside estradiol plus progestin; no autophagy manipulation or direct RIF validation.	(9, 10)
Timed and spatially resolved human RIF cohorts	RIF separated into a timing-displaced class and a hyperinflammatory class, with compartment-specific epithelial, stromal and immune change.	IFNG-dependent attachment effects *in vitro*; region-specific expression differences within CD56-positive areas.	Modest cohorts; cell proportions and inferred communication do not establish uNK function or causality.	(13, 50)

Functional claims apply only to the stated model. H3K9me3, histone H3 lysine 9 trimethylation.

On their own, changes in autophagy or uNK counts do not establish endometrial pathology. Endometrial CD56-positive counts vary substantially across studies, correlate poorly with peripheral counts, and do not consistently predict pregnancy outcome, supporting the ESHRE recommendation against routine NK-cell testing in RIF (1, 4). A systematic review of conventional and modern endometrial receptivity markers likewise found no established predictive value in clinical practice (58). The evidence is also insufficient to use the proposed links as a basis for autophagy-modifying or NK-directed treatment. Rapamycin remains an experimental tool: in abortion-prone mice, low and high doses produced opposing pregnancy outcomes (34). These findings do not establish efficacy or safety in preimplantation RIF. Prospective studies could examine whether progesterone responses in specific endometrial cell types are associated with directly measured autophagic flux. This requires clearly defined RIF cohorts, documentation of embryo competence in preceding transfers, and sampling standardized to cycle phase. Where feasible, longitudinal sampling would support this analysis alongside complementary flux assays and spatial confirmation. In patient-matched stromal-epithelial-uNK assembloids, graded increases or decreases in flux could be used to test effects on decidualization, uNK residency, and uNK function, followed by genetic rescue to assess reversibility. Blocking and add-back experiments would help identify which stromal or epithelial mediators are necessary and sufficient to connect progesterone and autophagy states with uNK behavior. Standardized uNK assays would measure senescent-cell clearance, secretory and angiogenic output, and tissue-residency programs. Their reproducibility and ability to predict implantation should be compared directly with CD56-positive counts, accounting for embryo characteristics and clinical covariates. Any resulting multimodal signature would need prespecified, registered multicenter validation in independent cohorts with continuous temporal sampling. It would have to add predictive value beyond embryo and clinical factors before it could guide treatment. Reproducible findings could then support intervention trials powered for reproductive and developmental outcomes, rather than implantation surrogates alone.

## 7 Conclusion

Autophagy may help connect steroid responsiveness and decidual cell state to epithelial competence, local uNK support, and decidual quality control (**Figure 1**). The evidence does not identify a consistent direction of flux change or establish a complete causal pathway from progesterone resistance through autophagy and uNK dysfunction to human implantation failure. Differences in cell type, cycle stage, stress, and assay choice may explain the contrasting results. Findings from preimplantation endometrium and RIF require separate interpretation from those in postimplantation decidua and RPL. Testing these hypotheses will require phase-resolved human sampling and direct flux measurements, combined with perturbation and rescue in multicellular models and uNK functional assays alongside cell counts. Prospective replication would then be needed to support the development of biomarkers or therapeutic targets. Routine NK testing is not recommended for RIF, and the proposed autophagy-immune links remain insufficient to guide treatment.

## Glossary

AKT: protein kinase B
ALDH1A3: aldehyde dehydrogenase 1 family member A3
ATG: autophagy-related
BMPR1A: bone morphogenetic protein receptor type 1A
BMP2: bone morphogenetic protein 2
cAMP: cyclic adenosine monophosphate
CDC42: cell division cycle 42
CXCL12: C-X-C motif chemokine ligand 12
CXCR4: C-X-C motif chemokine receptor 4
dNK: decidual natural killer
dpc: days post coitum
eGFP: enhanced green fluorescent protein
EOMES: eomesodermin
ESHRE: European Society of Human Reproduction and Embryology
FIP200: focal adhesion kinase family-interacting protein of 200 kDa
FOXO1: forkhead box O1
GSK3beta: glycogen synthase kinase 3-beta
H3K9me3: histone H3 lysine 9 trimethylation
HAND2: heart and neural crest derivatives expressed 2
HIF-1alpha: hypoxia-inducible factor 1-alpha
HOXA10: homeobox A10
HVEM: herpesvirus entry mediator
HYAL2: hyaluronidase 2
IFNG: interferon gamma
IGFBP1: insulin-like growth factor-binding protein 1
IL-15: interleukin 15
IVF: *in vitro* fertilization
LAMP2: lysosome-associated membrane protein 2
LC3: microtubule-associated protein 1 light chain 3
LH: luteinizing hormone
MITF: melanocyte inducing transcription factor
MMP9: matrix metalloproteinase 9
mTOR: mechanistic target of rapamycin
NK: natural killer
NNMT: nicotinamide N-methyltransferase
NOTCH1: Notch receptor 1
PAPSS2: 3’-phosphoadenosine 5’-phosphosulfate synthase 2
PGR: progesterone receptor
pNK: peripheral-blood natural killer
PRL: prolactin
RIF: recurrent implantation failure
RPL: recurrent pregnancy loss
SMAD1/5/9: SMAD family member 1/5/9
SQSTM1: sequestosome 1
T-HESCs: telomerase-immortalized human endometrial stromal cells
TNF: tumor necrosis factor
TNFRSF14: tumor necrosis factor receptor superfamily member 14
TNFSF14: tumor necrosis factor superfamily member 14
uNK: uterine natural killer
